# Tackling malignant melanoma epigenetically: histone lysine methylation

**DOI:** 10.1186/s13148-018-0583-z

**Published:** 2018-11-22

**Authors:** Elias Orouji, Jochen Utikal

**Affiliations:** 1 Department of Genomic Medicine, University of Texas MD Anderson Cancer Center, 1901 East Rd. South Campus Research Building 4, Houston, TX 77054 USA; 2 Skin Cancer Unit, German Cancer Research Center (DKFZ), Heidelberg, Germany; 3 Department of Dermatology, Venereology and Allergology, University Medical Center Mannheim, Ruprecht-Karl University of Heidelberg, Mannheim, Germany

**Keywords:** Histone methylation, Melanoma, Epigenetics, Histone demethylase, Histone methyltransferase, Small molecule inhibitor

## Abstract

Post-translational histone modifications such as acetylation and methylation can affect gene expression. Histone acetylation is commonly associated with activation of gene expression whereas histone methylation is linked to either activation or repression of gene expression. Depending on the site of histone modification, several histone marks can be present throughout the genome. A combination of these histone marks can shape global chromatin architecture, and changes in patterns of marks can affect the transcriptomic landscape. Alterations in several histone marks are associated with different types of cancers, and these alterations are distinct from marks found in original normal tissues. Therefore, it is hypothesized that patterns of histone marks can change during the process of tumorigenesis.

This review focuses on histone methylation changes (both removal and addition of methyl groups) in malignant melanoma, a deadly skin cancer, and the implications of specific inhibitors of these modifications as a combinatorial therapeutic approach.

## Background

### Histone modifications

**Fig. 1:**
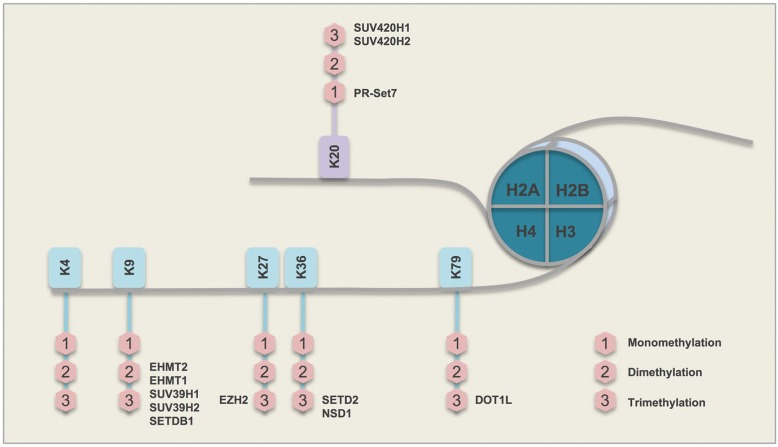
Major histone H3 and H4 lysine methyltransferase enzymes (H3K4, H3K9, H3K27, H3K36, H3K79, and H4K20) and their common sites of action

The nucleosome, the fundamental structural unit of chromosomes, is composed of two copies each of four core histones (i.e., H2A, H2B, H3, and H4), around which 147 bp of DNA is coiled [1, 2]. N-terminal tails of histone polypeptides can be altered by a variety of post-translational modifications, including methylation, acetylation, phosphorylation, ubiquitylation, glycosylation, ADP-ribosylation, carbonylation, and SUMOylation (collectively known as histone modifications) [3–6]. The acetylation of histones is controlled by the balanced action of histone acetyltransferases (HATs) and histone deacetylases (HDACs). Acetylated histones have been associated with actively expressed genes. However, histone methylation may have both repressive (H3K9, H3K27) or enhancing (H3K4) effects on transcription, depending on the residue that is modified [7]. Histone methylation can occur on lysine and/or arginine residues. Lysine residues at various positions along the histone N-terminal tail are common sites for methylation (Fig. 1). Lysine (K) methylation occurs by stepwise addition of one to three methyl groups, which can lead to unique functions of a genomic region. This stepwise conversion, from an unmethylated to a trimethylated lysine residue, is facilitated by histone methyltransferases (writers), and the backward demethylation process is catalyzed by histone demethylases (erasers). Further, a group of proteins (readers) recognize methyl-lysines throughout the genome [8]. Epigenetic changes along with genetic alterations can determine cell fate, either to maintain cell homeostasis or to promote tumorigenesis. Because epigenetic changes are reversible, understanding such changes is of crucial significance for drug development and exploring therapeutic strategies [9].

Several histone methylation alterations are known to play a role in the transition of melanocytes to melanoma cells. These alterations are the consequence of deregulation of their corresponding enzymes. In this review, we focus on major erasers and writers of histone lysine methylation as regulated via two categories of demethylase and methyltransferase enzymes and discuss compounds available to target these enzymes and reverse their impact.

## Main body

### Histone demethylases

#### JARID1B (H3K4-demethylase)

**Fig. 2:**
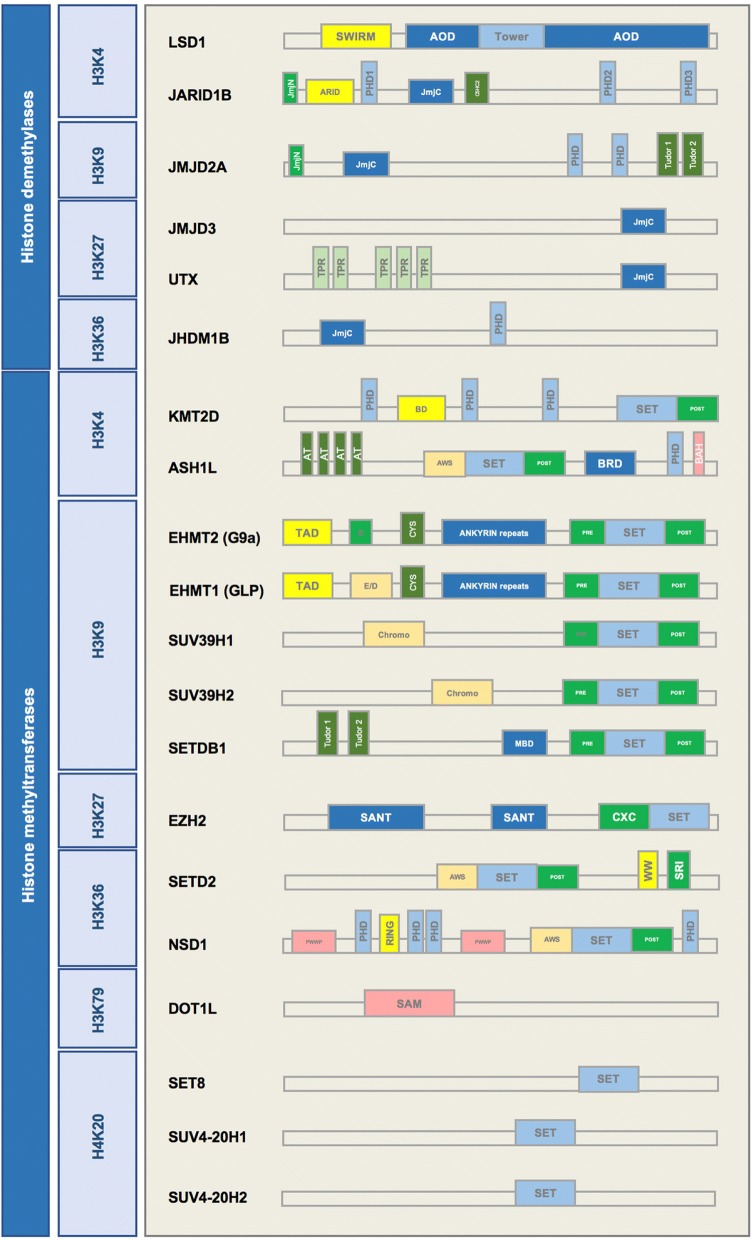
Schematic structures of histone lysine demethylases (H3K4, H3K9, H3K27, H3K36), histone lysine methyltransferases (H3K4, H3K9, H3K27, H3K36, H4K20) with SET domain, and histone methyltransferases without the SET domain (H3K79)

**Fig. 3:**
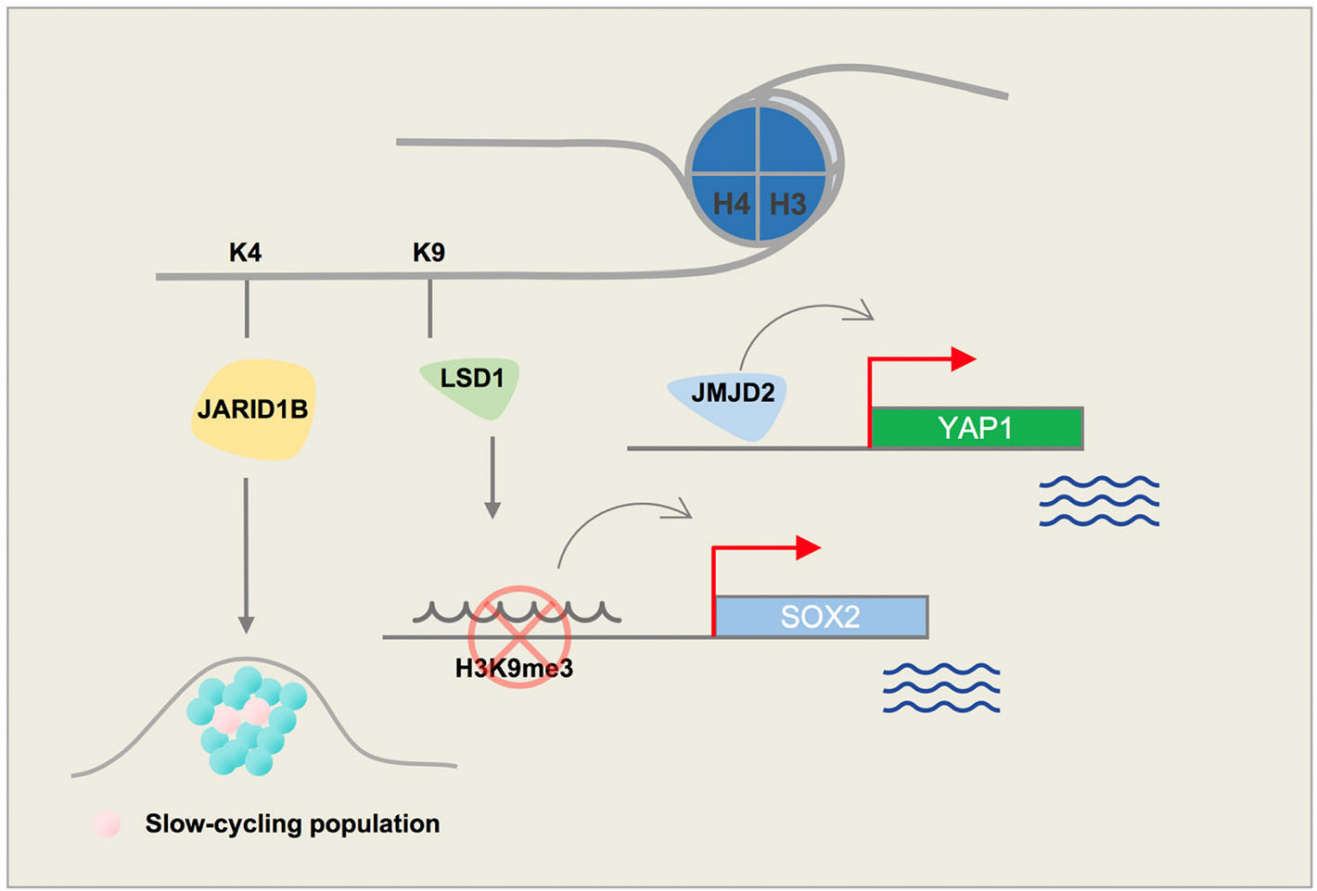
Schematic figure demonstrating key roles of histone lysine demethylases (H3K4 and H3K9) in melanoma

Several enzymes demethylate histone 3 at the position 4 lysine residue. One such enzyme, H3K4 demethylase JARID1B (PLU-1, KDM5B), plays a role in the development of melanoma, and regulates gene transcription and cell differentiation [10]. It has three plant homeodomain (PHD) finger domains in its structure (Fig. 2). PHD fingers are approximately 50–80 aa long and are usually involved in chromatin-mediated gene regulation. Two of these PHD fingers in JARID1B can bind to histones. JARID1B binds to its substrate through distinct PHD fingers. Finger PHD1 is highly specific for unmethylated H3K4 (H3K4me0). PHD3 is more specific to H3K4me3 [11]. The H3K4 demethylase JARID1B is highly expressed in nevi but not in melanoma; however, it marks a small subpopulation of melanoma cells that can cycle very slowly throughout the tumor mass and is essential for continuous tumor growth [12, 13] (Fig. 3). Interestingly, different treatments, including BRAF inhibitors (e.g., vemurafenib) as well as cytotoxic agents (e.g., cisplatin), lead to enrichment of slow-cycling, long-lasting melanoma tumor cells that express H3K4-demethylase JARID1B [14]. Eliminating this subpopulation of tumor cells might help overcome resistance to conventional treatments.

Treatment of melanoma tumors with MAPK inhibitors increases the H3K4 demethylase JARID1B-positive subpopulation of melanoma cells [15]. These cells are slow-cycling and treatment-resistant. Thus, in addition to curbing tumor growth with MAPK inhibitor, a supplementary agent is needed to effectively treat this subpopulation of cells. Several compounds inhibit JARID1B activity. KDM5-C49 is a potent inhibitor of the JARID1 enzyme family; however, its cellular permeability is limited. Therefore, KDM5-C70 was developed to be used in cellular assays and in vivo [16]. KDM5-C70 inhibits myeloma cells, increasing levels of H3K4me3 at the whole genome level [16]. 2,4-PDCA (2,4-pyridinedicarboxylic acid) acts in vitro to inhibit JARID1B and KDM4 demethylases. A high throughput screen of > 15,000 compounds conducted to identify inhibitors of JARID1B confirmed the activity of 2,4-PDCA and also revealed a novel compound, 2-4(4-methylphenyl)-1,2-benzisothiazol-3(2H)-one (PBIT) that can effectively inhibit JARID1B in vitro with no activity toward H3K27 demethylases, UTX or JMJD3 [17]. KDOAM-25 was recently introduced as a potent and selective JARID1 inhibitor with the ability to impact multiple myeloma cells [18].

#### LSD1 (H3K4-demethylase | H3K9-demethylase)

H3K4 methyl groups can also be removed by members of the LSD (lysine-specific histone demethylase) family [19, 20]. LSD1, also known as KDM1A, demethylates histone 3 on lysine residues at positions 4 and 9 (H3K4 and H3K9). LSD1 specifically removes 1 or 2 methyl groups from H3K4me2, converting it to H3K4me1/0. Monomethylation of H3K4 is a histone modification that marks enhancers throughout the genome. The histone demethylase role of LSD1 could change the gene transcription status by generating a H3K4me1 mark and activating enhancers, or by removing another methyl group and generating unmethylated H3K4. LSD1 has a SWIRM domain and 2 amino oxidase domains (AODs) in its structure (Fig. 2). A SWIRM domain is usually present in proteins with a role in chromatin remodeling and in expression of genes such as SMARC2 and SWI3.

LSD1 can bind to both gene promoters and distal elements. This feature of LSD1 is in line with its demethylation role on H3K4 (promoter) as well as H3K9 (distal) [21]. Trimethylated H3K9 is a well-known heterochromatin mark, which often highlights closed chromatin regions and is present on distal regions of genes. H3K9me3-active demethylase LSD1 is reported to disable senescence in melanocytes. Senescence halts the proliferation of melanocytes and further progression to melanoma cells; this role of LSD1 may cause Ras/Braf-induced transformation. Yu Y. et al. showed that enforced LSD1 expression in vivo can promote BRAF^V600E^-driven melanomagenesis [22]. Differentiated melanoma cells have an elevated level of H3K9 methylation. The presence of this repressive histone mark at the promoter region of pluripotency marks (e.g., SOX2) can inhibit expression that is essential to maintaining self-renewal and tumorigenicity. The H3K9 demethylase activity of LSD1 can epigenetically control SOX2 transcription and thus maintain cancer stem cell pluripotency [23, 24] (Fig. 3).

Depletion of histone demethylase LSD1 in cancer cells increases repetitive element expression that can stimulate anti-tumor T cell immunity and inhibit tumor growth [25]. LSD1 inhibition also suppresses colony formation and the growth of melanoma xenografts; LSD1 has not yet been pharmacologically targeted for treatment of melanoma [26].

Several known inhibitors of LSD1 have been identified, one of which is GSK2879552. This small molecule is an irreversible, selective, orally bioavailable inhibitor of LSD1. Anti-proliferative effects of this inhibitor have been reported in small cell lung carcinoma (SCLC) [27]. LSD1 interacts with pluripotency factors in human embryonic stem cells (hESCs) and is also critical for hematopoietic differentiation. Several studies have reported on effects of LSD1 inhibitors on hematological malignancies. In acute myeloblastic leukemia (AML) cell lines, GSK2879552 treatment inhibited proliferation [28, 29]. Treatment of neuroblastoma or breast cancer cell lines with tranylcypromine (TCP), another LSD1 inhibitor, or its analogs also resulted in the inhibition of cell proliferation [30]. Although LSD1 inhibition does not affect global levels of H3K4me1/2, its impact is observed near the TSS of LSD1 target genes. In GBM cell lines, the combination of TCP and an HDAC inhibitor led to a synergistic increase of apoptosis [30]. Recently, compound S2101, a new LSD1 inhibitor, is shown to have a greater effect than TCPs. Several other LSD1 inhibitors including T-3775440, NCD38, and RN1 are in pre-clinical stages of investigation [31–34].

#### JMJD2A (H3K9-demethylase | H3K36-demethylase)

H3K9/36me3 lysine demethylase JMJD2A (KDM4A) overexpression leads to copy gain of certain loci in chromosomes without global chromosome instability. JMJD2A-amplified tumors demonstrate copy gains for these regions [35]. Histone demethylase JMJD2A is involved in regulation of cell proliferation, interacts with RNA polymerase I, and is associated with active ribosomal RNA genes. Cellular localization of JMJD2A is controlled through the PI3K signaling pathway [36]. JMJD2A upregulation is observed in various cancers including the breast, colon, lung, and prostate [37–40]. Its overexpression in prostate cancer is positively correlated with tumor metastasis. ETV1 recruits JMJD2A to the YAP1 (a Hippo pathway component) promoter, leading to changes in histone lysine methylation in prostate cancer cells [40]. Yap1 also promotes melanoma metastasis through its TEAD-interaction domain [41]. A potential mechanism for this activity could be recruitment of JMJD2A to the Yap1 promoter, thus modulating its methylation (Fig. 3). Interestingly, another member of the JMJD2 family, histone demethylase JMJD2C, is increased in a subset of melanoma patients and promotes growth of aggressive tumors with metastasis [22].

#### JMJD3 (H3K27-demethylase)

H3K27 demethylase JMJD3 (KDM6B) plays a key role in transcriptional elongation and cell differentiation. Complexes of JMJD3 and proteins involved in transcriptional elongation, demethylate H3K27me3 on their target genes. This demethylation can abolish transcriptional repression of genes initially maintained by a H3K27me3 mark. JMJD3 modulates melanoma tumor microenvironment and promotes tumor progression and metastasis. JMJD3 does not alter proliferation in melanoma cells but does enhance other tumorigenic features of melanoma cells such as clonogenicity, self-renewal, and trans-endothelial migration. JMJD3 also promotes angiogenesis and macrophage recruitment [42]. Finally, JMJD3 can neutralize polycomb-mediated silencing at the INK4b-ARF-INK4a locus in melanoma. JMJD3 expression is upregulated in melanocytic nevi in response to oncogenic RAS signaling that leads to oncogenic-induced senescence [43, 44].

#### UTX (H3K27-demethylase)

Another mechanism of tumorigenesis is the activity of polycomb repressive complex 2 (PRC2). Any disruption in this complex can inhibit this process. Mutations in histone methyltransferase MLL3 (a subunit of the COMPASS complex with H3K4me1 methyltransferase activity) or BAP1 (a tumor suppressor) in cancer cells can inhibit H3K27 demethylase UTX and MLL3 recruitment to gene enhancers. Thus, inhibition of H3K27 methyltransferase PRC2 in these cells can restore normal transcription patterns [45]. A recent study proposed a mechanism for the role of UTX in suppressing myeloid leukemogenesis through deregulation of ETS and GATA programs [46]. H3K27-demethylase UTX activates gene transcription in melanoma at sites with poised promoters that are marked with trimethylated H3K27. Recruitment of UTX and another activating histone modifier, P300, is promoted through MEK1-mediated phosphorylation of RNF2 [47], indicating that UTX-mediated histone demethylation is a histone modification that may activate melanomagenesis.

GSK-J1 is a selective inhibitor of H3K27 demethylases JMJD3 and UTX and is inactive against a panel of other demethylases in JMJ family [48]. GSK-J4 is an ethyl ester pro-drug produced by masking the polarity of acidic groups of GSK-J1. GSK-J4 administration increases total nuclear H3K27me3 levels in cells [48]. High throughput screening of epigenetic compounds revealed sensitivity of neuroblastoma tumor cells to GSK-J4, a dual inhibitor of H3K27 demethylases UTX and JMJD3 [49].

#### JHDM1B (H3K36-demethylase | H3K79-demethylase)

JHDM1B (KDM2B), which is a known H3K36 demethylase member of the Jumonji C family of proteins, also plays a role as an H3K79 demethylase and a transcriptional repressor via SIRT1-mediated chromatin silencing [50]. JHDM1B specifically recognizes non-methylated DNA in CpG islands and recruits the polycomb repressive complex 1 (PRC1) that is required for sustaining synovial sarcoma cell transformation [51, 52]. JHDM1B is found to be highly expressed in glioblastoma compared to normal brain tissue and regulates the apoptotic response of GBM cells to TNF-related apoptosis-inducing ligand [53]. JHDM1B also plays a major role early in the reprogramming process for generation of induced pluripotent stem cells [54]. JHDM1B contributes to embryonic neural development by regulating cell proliferation and cell death [55] and plays a key role in cellular senescence and tumorigenesis [56].

A concise schematic picture of the role of major histone lysine demethylases that are involved in melanoma is provided in Fig. 3, and a list of these enzymes along with their known inhibitors are shown in Table 1.

**Table 1:** Histone lysine demethylases (KDMs) and their selective inhibitors

	Enzyme	Alias		Structure	Inhibitor	Reference
H3K4	LSD1	KDM1A	AOF2	SWIRM/AOD	GSK2879552 Tranylcypromine (TCP) Compound S2101 T-3775440 NCD38 OG-L002 RN1	[24–27]
	JARID1A	KDM5A	RBP2	PHD/ARID/ZF		
	JARID1B	KDM5B	PLU-1	PHD/ARID/ZF	KDM5-C49 KDM5-C70 2,4-PDCA PBIT KDOAM-25	[13–15]
	JARID1C	KDM5C	SMCX	PHD/ARID/ZF		
	JARID1D	KDM5D	SMCY	PHD/ARID/ZF		
H3K9	LSD1	KDM1A	AOF2	SWIRM/AOD		
	JMJD1A	KDM3A	JHDM2A	JmjC		
	JMJD1B	KDM3B	JHDM2B	JmjC		
	JMJD2A	KDM4A	JHDM3A	PHD/Tudor	2,4-PDCA	[14]
	JMJD2B	KDM4B	JHDM3B	PHD/Tudor		
	JMJD2C	KDM4C	JHDM3C	PHD/Tudor		
H3K27	UTX	KDM6A		TPR/JmjC	GSK-J1 GSK-J4	[41, 42]
	JMJD3	KDM6B		JmjC		
H3K36	FBXL11	KDM2A	JHDM1A	PHD/ZF/LRR		
	FBXL10	KDM2B	JHDM1B	PHD/ZF/LRR		
	JMJD2A	KDM4A	JHDM3A	PHD/Tudor		
	JMJD2B	KDM4B	JHDM3B	PHD/Tudor		
	JMJD2C	KDM4C	JHDM3C	PHD/Tudor		
H4K20	LSD1n		neuroLSD1			

### Histone methyltransferases

#### H3K4 methyltransferases

**Fig. 4:**
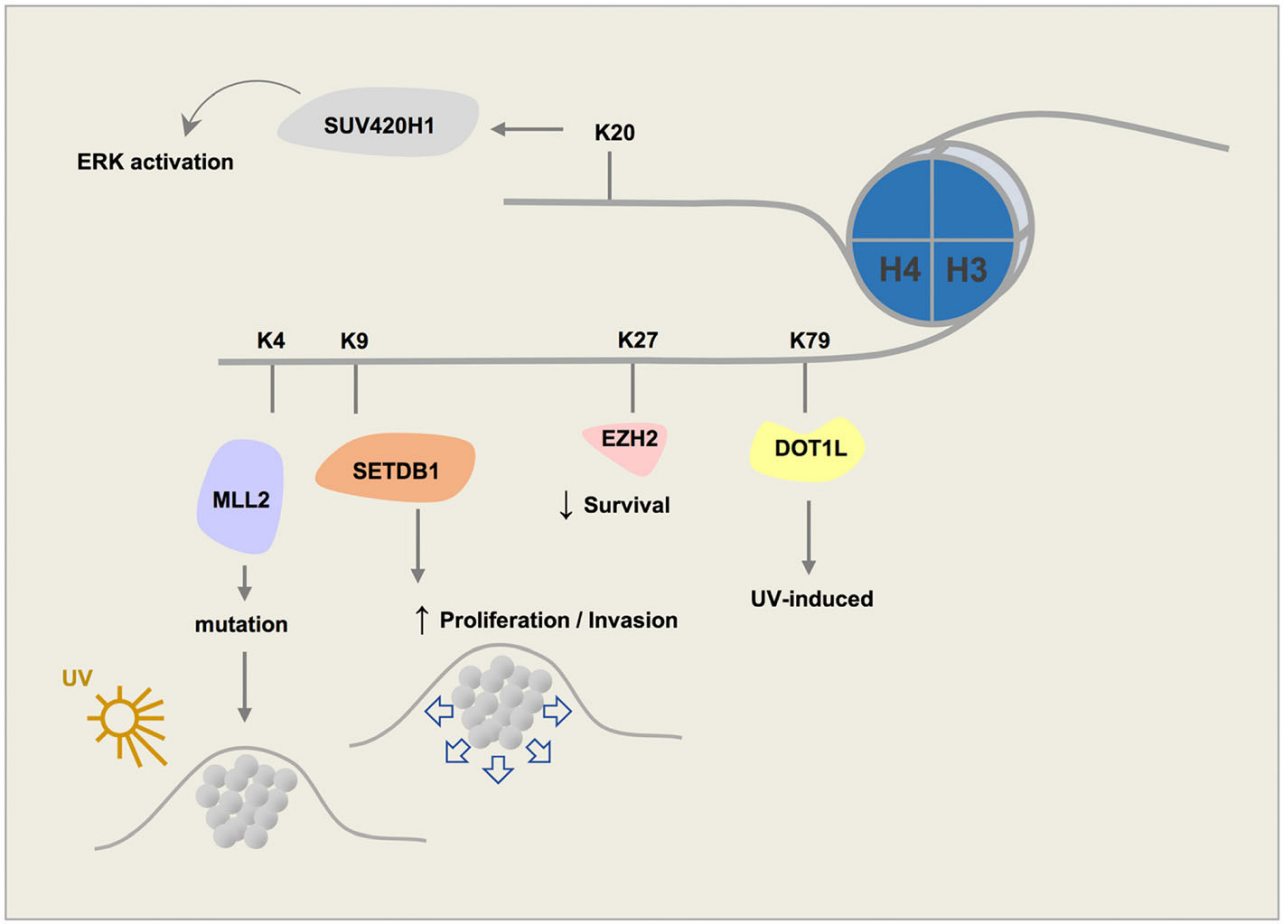
Schematic figure demonstrating key roles of histone lysine methyltransferases involved in the tumorigenesis and survival of melanoma

*KMT2D (MLL2)*. Monomethylation of H3K4 is a widely known feature of enhancers and gene promoters [57]. In mammals, KMT2D is part of a huge complex that induces monomethylation of promoter/enhancer regions [58, 59]. KMT2D is a member of the histone lysine methyltransferase (HKMTase) family that can induce genome accessibility and transcription and promotes an open chromatin state. The Cancer Genome Atlas (TCGA) data reveal that KMT2D is mutated in 15% of melanoma patients. Despite no clear association in TCGA cases between KMT2D and conventional subtypes of melanoma such as NRAS or BRAF, a subsequent study showed that KMT2D deregulates specific enhancers and genes in NRAS-mutant melanoma [60]. KMT2D is encoded by genes with the highest number of UVB signature mutations [61] (Fig. 4).

H3K4me2 is also an active histone mark enriched in sites toward the 5′-end of transcribing genes and present in several melanoma cell lines. An increase in the level of H3K4me2 has been observed in melanoma samples compared to normal skin of the same patients [62]. Interestingly, H3K4me2 levels were found to be lower in metastatic melanoma compared to primary tumors [63].

H3K4me3 is generally associated with promoters of actively transcribing genes [64, 65] or with genes that are poised for activation. H3K4me3 is linked to promoter activity. Global changes in H3K4me3 were observed in zebrafish when comparing melanoma and normal skin [66]. Comparison of metastatic to primary melanoma revealed that decreased levels of H3K4me3 in metastases are significantly associated with repressed expression of genes in embryonic stem cells (ESCs), including targets of PRC. Further, increased levels of H3K4me3 are associated with interferon and inflammatory response genes [59]. Finally, HDAC inhibitors that promote an open chromatin state also increase H3K4 trimethylation in vitro [67].

*ASH1L (ASH1, KMT2H)*. ASH1L is an H3K4/H3K36 methyltransferase. Nucleotide excision repair (NER) is a known mechanism to prevent formation of UV-induced melanoma tumors. The NER mechanism is in place upon recruitment of H3K4 histone methyltransferase ASH1L [68]. Loss of Ash1l leads to increased proliferation of keratinocytes [69]. ASH1L can act as an H3K36 dimethyltransferase to recruit MLL to chromatin and maintain hematopoiesis. This action supports a role of ASH1L in MLL-mediated leukemia oncogenesis and indicates that it could be a target for leukemia therapy [70].

WDR5 is an essential part of the KMT2 (MLL) complex that trimethylates H3K4. An interaction exists between this protein and the catalytic domains of KMT2. Despite the lack of chemical compounds that directly target the SET domain in KMT2 complexes, several compounds can specifically disrupt the interaction between WDR5 and MLL [71]. MM-102 specifically targets MLL-WDR5 interaction, inhibits cell growth, and induces apoptosis in leukemia cells [72]. MM-401 also specifically inhibits MLL activity by blocking MLL-WDR5 interaction [73]. A third compound, WDR5-0103, antagonizes the interaction of WDR5 with MLL by competing with MLL for their mutual binding site on WDR5 [74].

#### H3K9 methyltransferases

Monomethylated H3K9 is shown to be enriched in the TSS of active genes [75]. Unlike H3K9me1, both di- and trimethylated H3K9 are often found in silenced genes; however, monomethylated H3K9 may also mark distinct regions of silent chromatin in the presence of a monomethylated H4K20 mark [76].

*EHMT*. Dimethylated H3K9 has a higher global level in melanoma samples compared to the normal skin tissue [62]. EHMT2 (G9A) mono- and dimethylates H3K9. Methylated H3K9 recruits HP1 proteins that cause transcriptional repression [77]. HP1 proteins harbor a methyl lysine binding chromodomain that binds to methylated H3K9 [78]. HP1 proteins in human cells include HP1α, β, and γ that play key roles in the formation of transcriptionally inactive heterochromatin. Interestingly, in several cancer types (not including melanoma), inhibition of EHMT2 resulted in the arrest of cancer cell proliferation and a decrease in cell survival [79]. Another study proposed that G9A contributes to ovarian cancer metastasis [80]. EHMT1 (GLP) is also a histone methyltransferase that specifically mono- and dimethylates H3K9 in euchromatin.

*SUV39*. Three known members in this family have been identified. SUV39H1 is a histone methyltransferase that specifically trimethylates H3K9me1 and plays a crucial role in establishment of constitutive heterochromatin at pericentromeric and telomeric regions. Formation of various cancer types including rhabdomyosarcoma and melanoma are sensitive to the loss of SUV39H1 [81]. In differentiated melanoma cells, silencing SUV39H1 or the other H3K9 methyltransferase, G9A, can elevate their self-renewal capabilities [24]. SUV39H2, a second member of the family, has a similar structure to SUV39H1 (Fig. 2). SUV39H2 may play a role in higher order chromatin structure during spermatogenesis [82, 83]. SUV39H2 trimethylates histone lysine demethylase LSD1 and prevents degradation of this protein due to polyubiquitination, thus stabilizing its structure in the cell [84]. SETDB1 (ESET) is the third member of the SUV39 family of proteins. It is overexpressed in several cancers including human melanomas [85]. SUV39 proteins, unlike the HP1 chromodomain of the EHMT complex that shows high affinity for dimethyl, prefer the trimethyl state [86]. Hence, histone lysine methyltransferase SETDB1 also primarily trimethylates H3K9. SETDB1 is known to accelerate melanoma progression [85] (Fig. 4). Previously, SETDB1 was reported to be highly amplified in melanoma and to be associated with a more aggressive phenotype through regulation of thrombospondin 1 [87].

Chaetocin is a competitive inhibitor of S-adenosyl-L-methionine (SAM) and was the first histone lysine methyltransferase inhibitor to be identified. It specifically inhibits lysine histone methyltransferases of the SUV39 family, making the compound useful in the study of heterochromatin-mediated gene repression [88]. BIX01294 is an inhibitor of GLP and G9A methyltransferase [89]. This compound does not compete with cofactor SAM but acts as a competitive inhibitor of N-terminal peptides of H3 [90]. It selectively impairs the generation of H3K9me2 in vitro. UNC0321 is a newer modification of BIX12094 with a greater potency against G9A and GLP [91].

#### H3K27 methyltransferases

*EZH2*. Unlike monomethylated H3K27, which is associated with gene activation, di-/trimethylated H3K27 act as repressive marks [75, 92]. Histone lysine methyltransferase EZH2 is mostly responsible for trimethylation of lysine 27 on histone 3 (H3K27me3). EZH2 is the catalytic subunit of the polycomb repressive complex 2 (PRC2). PRC2 has 3 other subunits EED, SUZ12, and RBBP4. PRC2 mediates gene repression through chromatin reorganization by methylating H3K27. Therefore, H3K27me3 is associated with repressed chromatin. Mutations in EZH2 could result from the replacement of a tyrosine in its SET domain (Tyr641). This change can reduce enzymatic activity of EZH2 resulting in a higher affinity to mono- or dimethylated H3K27 [93]. In addition, in various melanoma cell lines, Tyr641 mutations of EZH2 have shown to alter the substrate specificity of EZH2 to H3K27me2, causing an increase in H3K27me3 and depletion of H3K27me2 [94]. H3K27me1/me2 produced by wild-type EZH2 could be methylated by Tyr641-mutant EZH2 to generate elevated levels of H3K27me3 that can promote tumorigenesis [95]. High levels of EZH2 activation are observed in neuroblastoma, hepatocellular carcinoma, small cell lung cancer, and melanoma [96]. Increased expression of EZH2 is associated with melanoma progression and decreased overall patient survival [97]. Protein levels of EZH2 increase incrementally from benign nevi to cutaneous malignant melanoma, which also suggests that EZH2 may play a role in the pathogenesis and tumorigenesis of melanoma [98–100] (Fig. 4). EZH2 suppresses senescence in melanoma by repressing CDKN1A expression independent of p16^INK4a^ expression or p53 function [98]. In addition, aggressive melanoma cells have excessive levels of H3K27me3 accompanied by EZH2 overexpression [101], in which EZH2 controls melanoma progression and metastasis by silencing tumor suppressors [102].

Several available EZH2 inhibitors have been used in numerous studies. EPZ-6438 (Tazemetostat) is an EZH2 inhibitor in phase 1 and 2 clinical trials. This drug has been investigated for multiple cancer types, including B cell non-Hodgkin lymphoma and advanced solid tumors [103]. EPZ011989 is a potent and orally bioavailable EZH2 inhibitor [104]. GSK126 is a potent and also highly selective inhibitor of EZH2 that decreases global H3K27me3 levels and reactivates silenced PRC2 target genes. GSK126 inhibits proliferation of EZH2-mutant diffuse large B cell lymphoma (DLBCL) cell lines and mouse xenografts [105]. EZH2 inhibition using this small molecule is proposed to increase NK cell death suggesting an immunosuppressive effect of EZH2 [106]. Treatment with another EZH2 inhibitor, GSK503, in a melanoma mouse model, blocked tumor growth and metastasis formation. In human melanoma cells, GSK503 impaired cell proliferation and invasiveness, along with re-expression of tumor suppressors associated with increased patients’ survival [102]. In osteosarcoma, GSK343, another inhibitor of EZH2, restricts cell viability and promotes apoptosis. GSK343 inhibits EZH2 expression and its substrate, H3K27me3. It also inhibits fuse binding protein 1 (FBP1) expression, a c-Myc regulator [107]. In cancers with genetic alteration in EZH2, such as various forms of lymphoma, EPZ005687 reduces H3K27 methylation. Mutations in EZH2 result in a dependency on its enzymatic activity for proliferation that could make EPZ005687 a treatment for cancers in which EZH2 is genetically altered [108].

Another approach to inhibit the PRC2 complex and subsequently H3K27 methylation is to block another component of this complex, such as EED. In a recent study, a compound, EED226, was developed to block PRC2. EED226 is a potent and selective PRC2 inhibitor that directly binds to the H3K27me3 binding pocket of EED. It induces regression of human lymphoma xenograft tumors [109, 110].

#### H3K36 methyltransferases

Di-/trimethylation of H3K36 is usually associated with transcriptional activation and double-strand break repair. H3K36me2 can be deposited near the breaks and can recruit early repair factors [111]. H3K36me3 is considered as a hallmark of active gene transcription. It is commonly enriched at the body of active genes and particularly at their 3′-terminal regions. Its activity produces a methylation pattern distinct from that of other histone methylations [112]. H3K36me3 is involved in defining exons which are enriched in nucleosomes. Nucleosomes are more frequent in areas with this particular histone mark [113]. H3K36 trimethylation is also shown to play a role in homologous recombination (HR) repair in human cells [114]. Another study proposes a less known role of H3K36me3 in combination with other histone marks in contributing to the composition of heterochromatin [115]. Also, H3K36 methylation antagonizes PRC2-mediated H3K27 methylation [116].

*SETD2*. SETD2 is the only known gene in human cells responsible for trimethylation of lysine 36 of Histone H3 (H3K36) [117]. This enzyme is mutated in 11.5% of renal clear cell carcinoma tumors [118]. The H3K36me3 mark is associated with DNA methylation in actively transcribed gene regions [119]. A TCGA study also suggests crosstalk between H3K36 methylation and DNA methylation. Renal clear cell carcinoma tumors with SETD2 mutations had DNA hypomethylation at non-promoter regions that are marked by H3K36me3 [118]. A similar pattern is found in sarcoma where H3K36 mutation prevents methylation as well as mesenchymal progenitor cell differentiation and induces sarcoma [120]. SETD2 counteracts Wnt signaling and signaling loss promotes intestinal tumorigenesis in vivo. Mechanistically, SETD2 downregulation affects alternative gene splicing leading to tumor inhibition [121].

*NSD1*. NSD1 binds near various promoter elements and interacts with H3K36 and RNA Pol II, to regulate transcription [122]. H3K36me2 methyltransferase NSD1 is a modulator of PRC2 function and demarcates H3K27me2 and H3K27me3 domains in ESCs [123]. NSD1 inactivating mutations define a hypomethylated subtype in different cancer types such as head and neck squamous cell carcinoma (HNSC) and lung squamous cell carcinoma (LUSC) [124–126]. This subtype is also characterized by low T cell infiltration into the tumor microenvironment, suggesting an identification of this subtype prior to immunotherapy [125]. NSD1 inactivation along with SETD2 mutation that causes a distorted H3K36 is a key feature of clear cell renal cell carcinoma [127]. NSD1 mutations may have a genome-wide impact on DNA methylation [128]. NSD1 expression in patient-derived metastatic cell lines is significantly higher compared to normal melanocytes; however, NSD1 upregulation does not apply to primary tumors developing to metastatic lesions [129].

N-propyl sinefungin (Pr-SNF) is shown to interact preferentially with SETD2 and act as an inhibitor of this protein [130]. However, no compounds that can target NSD proteins selectively have been identified. Due to the high similarity of the SET domain of NSDs to that of GLP and G9A, BIX01294 would be capable of inhibiting H3K36 methyltransferase NSD1/2/3 [131].

#### H3K79 methyltransferases

*DOT1L.* H3K79 is a histone mark associated with active chromatin and transcriptional elongation [132]. DOT1L is the only enzyme identified that methylates H3K79. Interestingly, DOT1L has no SET domain in its protein structure (Fig. 2). It is linked to both active and repressed genes. However, H3K79 methylation is mainly a mark of active transcription. DOT1L methylates at 3 levels, mono-di-, and trimethylation. H3K79 methylation has a crucial role in heterochromatin formation and chromosome integrity [133]. An oncogenic role of DOT1L histone H3 lysine 79 (H3K79) methyltransferase in MLL-rearranged leukemogenesis has been established. Unlike leukemia, DOT1L plays a repressive role in UV-induced melanoma development. DOT1L is frequently mutated in human melanoma, leading to a reduced level of H3K79 methylation. DOT1L depletion will cause UV-induced DNA damage not to be efficiently repaired, thus encouraging progression of melanoma [134] (Fig. 4).

Pinometostat (EPZ5676) is a selective, small molecule inhibitor of DOT1L. MLL-rearranged cells and xenograft models treated with this inhibitor showed reduced levels of H3K79me2 [135, 136]. This drug is currently in phase 1 clinical trials and has modest clinical activity [137, 138]. EPZ004777 is a specific, SAM-competitive inhibitor of DOT1L. It selectively kills leukemia cells with MLL rearrangements. A chemical analog of this drug, SGC 0946, with improved solubility and potency has also been developed [139].

#### H4K20 methyltransferases

*PR-Set7 (SET8)*. H4K20me1 is linked to transcriptional activation, a modification that is present in highly transcribed genes. Histone H4 lysine methyltransferase PR-Set7 (SET8) mono-methylates H4K20 [140, 141]. This enzyme plays a role in several processes including DNA damage response, chromatin compaction, DNA replication, transcriptional regulation, and tumorigenesis [140, 142]. Loss of SET8 could cause cell cycle defects and promote DNA damage [143]. Depletion of SET8 in melanoma cells under treatment with the NEDD inhibitor, pevonedistat, indicated that preventing degradation of SET8 is essential for effective impact of this drug in melanoma suppression because of its role in DNA re-replication and senescence [144].

*SUV420H1/H2*. Dimethylation of H4K20 is the most abundant methylation state and is present in about 80% of H4 histones [145]. H4K20me2 plays a role in cell cycle control and DNA damage response whereas H4K20me3 takes part in transcriptional repression and is a hallmark of silenced heterochromatin regions. Loss of trimethylated H4K20 is considered a common mark of cancer. Both these methyltransferases have a Zn-binding post-SET domain [146]. SUV420H1 and SUV420H2 play roles in NHEJ-directed DNA repair by di- and trimethylation of H4K20 [146]. Further, overexpression of SUV420H1 may lead to activation of ERK through enhancement of ERK phosphorylation and transcription [147] (Fig. 4).

A-196 is a selective inhibitor of SUV420H1 and SUV420H2. This drug induces a genome-wide decrease in H4K20me2/me3 and increases H4K20me1 [148].

A concise schematic picture of the role of major histone lysine methyltransferase involved in melanoma is provided in Fig. 4, and a list of these enzymes along with their known inhibitors is shown in Table 2.

**Table 2:** Histone lysine methyltransferases (KMTs) and their selective inhibitors

	Enzyme	Alias		Structure	Inhibitor	Reference
H3K4	MLL1	KMT2A	TRX1	SET/PHD/FYRC/BD	MM102* MM-401* WDR5-0103*	[66–68]
	MLL2	KMT2B	MLL4	SET/PHD/FYRC/BD		
	MLL3	KMT2C	HALR	SET/PHD/FYRC		
	MLL4	KMT2D	MLL2	SET/PHD/FYRC		
	MLL5	KMT2E		SET/PHD		
	SET1A	KMT2F	SETD1A	SET/RRM		
	SET1B	KMT2G	SETD1B	SET/RRM		
	ASH1L	KMT2H	ASH1	SET/PHD		
	SET7/SET9	KMT7	SETD7	SET		
H3K9	G9A	KMT1C	EHMT2	SET/TAD/ANK	BIX01294 UNC0321	[83, 85]
	GLP	KMT1D	EHMT1	SET/TAD/ANK		
	SUV39H1	KMT1A	H3-K9-HMTase 1	SET/CD	Chaetocin	[82]
	SUV39H2	KMT1B	H3-K9-HMTase 2	SET/CD		
	SETDB1	KMT1E	H3-K9-HMTase 4	SET/Tudor/MBD		
	RIZ1	KMT8	PRDM2	PR/ZF		
H3K27	EZH2	KMT6		SET/SANT	EPZ-6438 (Tazemetostat) EPZ011989 GSK126 GSK503 GSK343 EPZ005687 CPI-1205 CPI-169 EI1 EED226**	[96–99, 101–104]
H3K36	SET2	KMT3A	SETD2	SET/SRI	N-propyl sinefungin (Pr-SNF)	[124]
	NSD1	KMT3B	STO	SET/PHD	BIX01294	[125]
	SMYD2	KMT3C	ZMYND14	SET/MYND		
	ASH1L	KMT2H	ASH1	SET/PHD/BD		
H3K79	DOT1L	KMT4		LRR	EPZ5676 (Pinometostat) EPZ004777 SGC 0946	[129–133]
H4K20	PR-Set7	KMT5A	SETD8	SET		
	SUV420H1	KMT5B	CGI85	SET	A-196	[142]
	SUV420H2	KMT5C		SET		
	NSD1	KMT3B	STO	SET/PHD		

*These compounds target MLL-WDR5 interaction

**This compound blocks EED, another component of PRC2 to inhibit H3K27 methylation

## Discussion

### Future direction

Despite the emergence of several new targeted therapies and immunotherapy drugs, tumor resistance to these novel therapies is a major hurdle that reflects a need for more efficient therapeutic strategies. Numerous studies have been published in recent years to clarify the role of histone methylation and associated enzymes in tumorigenesis. Because of the reversible nature of histone methylation, demethylase and methyltransferase enzymes as well as chemical compounds that inhibit the activities of the enzymes and can therefore change the level of methylation at specific histone sites are under comprehensive investigation as targets for cancer therapy. Current understanding of the role of these enzymes suggests that they regulate transcription activation and repression based on the histone involved and the specific amino acid residue being modified.

Various compounds that target the epigenome, such as HDAC inhibitors (e.g., the entinostat, NCT00185302) or DNMT inhibitors (e.g., the decitabine, NCT00030615), have been used in clinical trials to treat melanoma patients, but the use of histone methylating/demethylating agents requires substantial additional investigation. As discussed in this review, different histone demethylases play a role in cell transformation, although H3K4 and H3K9 demethylases are most consistently reported [12–14, 22–24]. This result could be due to an insufficient body of evidence for lesser known erasers, but investigation of JARID1 or LSD1 inhibitors in extensive in vivo experiments, and eventually, in clinical trials, could be of clinical significance.

A different approach to clinical application of histone demethylase function might be used in these enzymes in removing methylation in regions of the genome that are unmethylated in normal cells. An enzyme recently used in this manner is LSD1. This enzyme was used in fusion with dCas9 to modulate histone methylation. It has been applied on certain gene enhancers in the genome to suppress specific gene transcription without disrupting local genomic architecture [149]. Lack of impact to the genomic structure is key because disruption of the chromatin structure can change DNA-protein interactions at the global level with unpredictable consequences. The use of such enzymes in combination with gene editing technology could allow development of novel treatment modalities involving removal of active histone marks on promoter regions of oncogenes or repressive histone marks on distal elements of tumor suppressor genes, both of which are common features seen particularly in cutaneous melanoma.

As we have summarized here, past literature leads us to several epigenetic patterns that seem to be frequently repeated in distinct types of cancer, including the role of histone methylation modifiers on the promoters of major known drivers of a particular type of cancer. These drivers (i.e., MITF, c-Myc in melanoma) have been recently associated with clusters of non-coding regions on the genome called super enhancers that are usually marked with enhancer marks such as H3K4me1 as well as H3K27ac [150]. Inactivation of these frequently methylated histone tails could help to restore cell homeostasis.

Lastly, epigenome-wide studies have partly elucidated the function of combinations of histone methylation marks in melanoma, although understanding of the melanoma epigenome is still unclear. These studies reveal that in a given region of DNA, more than one histone methylation mark could be active in regulating gene expression. Further, the 3D structure of the genome can juxtapose certain regions of the DNA harboring specific histone marks and other regions, thereby activating or repressing genes that may be mega-bases down or upstream.

As per the available information, a comprehensive therapeutic approach, involving minimal disruption of the genome architecture, seems to be essential. Such a therapeutic strategy could help reverse or halt the function of a combination of histone modifications that lead to cell transformation.

## Abbreviations

AOD: Amino oxidase domain
DLBCL: Diffuse large B cell lymphoma
ESC: Embryonic stem cell
ESET: ERG-associated protein with SET domain
EZH2: Enhancer of zeste homolog 2
HAT: Histone acetyltransferase
HDAC: Histone deacetylase
HKMTase: Histone lysine methyltransferase
HNSC: Head and neck squamous cell carcinoma
LUSC: Lung squamous cell carcinoma
MAPK: Mitogen-activated protein kinase
NER: Nucleotide excision repair
NHEJ: Non-homologous end joining
PHD: Plant homeodomain
PRC: Polycomb repressive complex
SAM: S-adenosyl-l-methionine
SCLC: Small cell lung carcinoma
TCGA: The Cancer Genome Atlas
TCP: Tranylcypromine
TSS: Transcription start site
